# Effect of short-term high-temperature exposure on the life history parameters of *Ophraella communa*

**DOI:** 10.1038/s41598-018-32262-z

**Published:** 2018-09-18

**Authors:** Hongsong Chen, Xingwen Zheng, Min Luo, Jianying Guo, Ghulam Sarwar Solangi, Fanghao Wan, Zhongshi Zhou

**Affiliations:** 1 State Key Laboratory for Biology of Plant Diseases and Insect Pests, Institute of Plant Protection, Chinese Academy of Agricultural Sciences, Beijing, 100193 China; 2 Guangxi Key Laboratory for Biology of Crop Diseases and Insect Pests, Institute of Plant Protection, Guangxi Academy of Agricultural Sciences, Nanning, 530007 China; 3 Department of Entomology, Sindh Agriculture University Sub Campus, Umerkot, 69100 Pakistan

## Abstract

Extreme heat in summer is frequent in parts of China, and this likely affects the fitness of the beetle *Ophraella communa*, a biological control agent of invasive common ragweed. Here, we assessed the life history parameters of *O*. *communa* when its different developmental stages were exposed to high temperatures (40, 42 and 44 °C, with 28 °C as a control) for 3 h each day for 3, 5, 5, and 5 days, respectively (by stage). The larval stage was the most sensitive stage, with the lowest survival rate under heat stress. Egg and pupal survival significantly decreased only at 44 °C, and these two stages showed relative heat tolerance, while the adult stage was the most tolerant stage, with the highest survival rates. High temperatures showed positive effects on the female proportion, but there was no stage-specific response. Treated adults showed the highest fecundity under heat stress and a similar adult lifespan to that in the control. High temperatures decreased the F_1_ egg hatching rate, but the differences among stages were not significant. Negative carry-over effects of heat stress on subsequent stages and progenies’ survival were also observed. Overall, heat effects depend on the temperature and life stage, and the adult stage was the most tolerant stage. *Ophraella communa* possesses a degree of heat tolerance that allows it to survive on hot days in summer.

## Introduction

Common ragweed, *Ambrosia artemisiifolia*, an invasive weed, is a nationwide problem with ecological and health costs in China^1^. It is usually found alongside roads and in crop fields and orchards^2^. It has gradually spread and can now be found in 21 provinces in China^1,3^. *Ophraella communa* originates from North America^4^; accordingly, in China, it is used as a biological control agent of *A*. *artemisiifolia*^1^, with both larvae and adults feeding on *A*. *artemisiifolia* leaves^1^. When the beetles occur at a high population size, they exert strong control of the invasive weed^5,6^, and they significantly reduce seed production, even if a few defoliated plants survive^7^. With the rapid expansion, broad dispersal, high productivity, high feeding amount^8^, and rapid evolution of *O*. *communa*^9^, the beetle has provided complete defoliation and prevented flowering and seed set in ragweed plants in Europe^10^. In recent years, this natural enemy has been reported in eastern^11^, central^12^, and southern parts of China^13^, and it has been shown to provide effective control of *A*. *artemisiifolia* in the field in China at some sites^1^.

Research on *O*. *communa* has provided important insights into temperature. Temperature is a dominant abiotic factor that strongly affects organisms’ behaviour, physiology, life history, distribution, and abundance^14^. Insects have an optimal temperature range to which their biological functions are best adapted; under supra-optimal temperatures, insects might incur physiological costs and suffer damage that lowers their performance^15^. Most insects have the ability to tolerate some degree of temperature fluctuation^16^, but lethal temperatures are usually between 40 and 50 °C depending on insect species and life stage^17^. Extreme heat in summer has become more frequent in recent years compared to in the early 20^th^ century in many regions around the world^18^. In many parts of China, summer maximum daily temperatures in the field often exceed 40 °C for several hours, and the number of such hot days has also increased in the last few years^19–21^. Heat shock affects the developmental fitness and behaviour of insects^22^. The effects of heat stress have been reported in a variety of insects, including *Trialeurodes vaporariorum*^23^, *Metopolophium dirhodum*^24^, *Plutella xylostella*^19^, *Cryptolaemus montrouzieri*^25^, and *Aphelinus asychis*^21^. Most of the effects were negative, as heat stress could affect insects’ behaviour, growth, development, reproduction, survival, and offspring fitness^26,27^. The effects of thermal stress depend on the frequency, amplitude, and duration of the stress^26^. Meanwhile, the effects of thermal stress differ based on which life stage experienced the heat stress^28^. Both basal tolerance and plastic responses contribute to the ability of ectotherms to counter heat stress^29^. Behavioural thermoregulation allows mobile stages (like larvae and adults) to escape lethal temperatures, and less mobile stages (like eggs and pupae) thus have to cope with and tolerate more extreme conditions than mobile stages^30^. According to this principle, immobile stages are expected to show higher basal thermotolerance than mobile stages^28–30^. Any developmental stage of insect species with relatively short generation periods may experience heat stress in the field, thus the population sizes of these species may be affected.

The optimal developmental temperature for *O*. *communa* found in one laboratory study ranged from 25 to 28 °C^31^. When the ambient temperature was ≥36 °C, all the first instar larvae died within 24 h, and the survival of other stages and female fecundity decreased significantly^3^. In the long summer in China, any developmental stage of *O*. *communa* may experience brief heat stress based on the relatively short generation period of this leaf beetle^31^. Our previous study showed that the pre-adult development and survival, adult survival, longevity and fecundity of *O*. *communa* were all adversely affected after 2 h of heat stress at ≥35 °C in the laboratory and that populations of the beetle may be significantly affected in summer days in southern China^3^. However, the previous study only focused on the rapid death caused by heat stress for 3 days at 2 h per day. In general, insects may die rapidly or more slowly until later stages of development at high temperatures^27^; thus, the effects of heat stress could carry over into the adult stage^32^ or even into the next generation^27^. The frequency and duration of extreme temperatures in China were recorded in the field as being more than 3 days in the developmental period of *O*. *communa* and longer than 2 h per day^19–21^. Therefore, the carry-over effects of high temperatures on *O*. *communa* and under true field conditions should be studied. Based on the extreme summer temperatures in central and southern China, the objective of the present study was to investigate the effects of short-term high-temperature exposure on the life history parameters (survival, female proportion [F/(F + M)], adult longevity, and F_1_ egg hatching rate [that of the subsequent generation]) of *O*. *communa* to provide valuable information for forecasting the population dynamics of this biocontrol agent in areas with periods of extreme heat.

## Results

### Effects of high temperatures on survival and female proportion [F/(F + M)]

**Figure 1:**
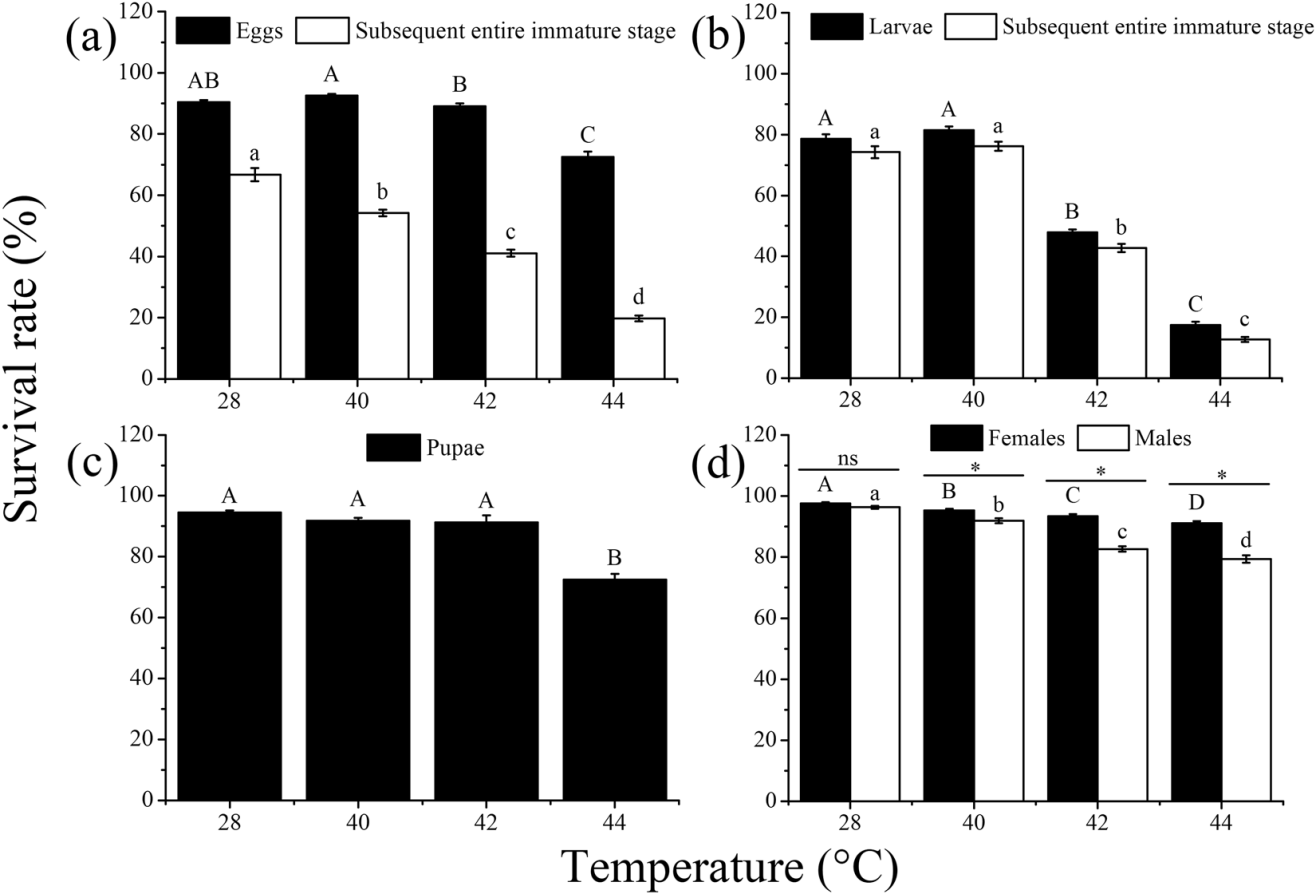
Mean ± SE survival rate (%) of *Ophraella communa* after exposure of eggs (**a**) larvae (**b**) pupae (**c**) and adults (**d**) to high temperatures [28 (control), 40, 42 and 44 °C] for 3 h each day for 3, 5, 5, and 5 days, respectively (by stage). Black columns represent the survival rate (%) of the individual treated stages, while white columns indicate the survival rate (%) over all relevant stages (or male adults). The sample sizes for each treatment with five replicates were (**a**) 2,000, (**b**) 1,800, (**c**) 1,100 and (**d**) 250 (125 males and females). Different upper-case letters indicate significant differences among temperatures for treated eggs, larvae, pupae, and female adults (Tukey’s HSD test, *P* < 0.05). Different lower-case letters indicate significant differences among temperatures for subsequent stages and male adults (Tukey’s HSD test, *P* < 0.05). *Indicates a significant difference between males and females within each temperature (Tukey’s HSD test, *P* < 0.05); ns, not significant.

High temperature, the developmental stage exposed, and their interaction all had a significant influence on the survival of *O*. *communa* (*P* < 0.05; Table 1). Overall, the survival rates of individuals of the leaf beetle were significantly affected by short exposures to high temperatures (40, 42, or 44 °C for 3 h) in eggs (*F*_3,16_ = 73.39; *P* < 0.0001), larvae (*F*_3,16_ = 474.03; *P* < 0.0001), and pupae (*F*_3,16_ = 44.99; *P* < 0.0001), and in the egg to adult (*F*_3,16_ = 224.83; *P* < 0.0001) and larva to adult periods (*F*_3,16_ = 439.82; *P* < 0.0001) (Fig. 1a–c). Although the survival of newly emerged adults was significantly affected by brief high temperatures (female: *F*_3,16_ = 21.04; *P* < 0.0001; male: *F*_3,16_ = 86.06; *P* < 0.0001), the survival rates of adults were still above 90% or close to 80% for females and males, respectively, at 44 °C (Fig. 1d). The survival rates of exposed females were significantly higher than those of males at 40 °C (*F*_1,8_ = 11.93; *P* = 0.0048), 42 °C (*F*_1,8_ = 79.00; *P* < 0.0001), and 44 °C (*F*_1,8_ = 81.72; *P* < 0.0001), but not at 28 °C (*F*_1,8_ = 3.66; *P* = 0.0798) (Fig. 1d). The adult stage proved to be the most heat-tolerant stage, with survival rates of 91.13% and 79.36% for females and males, respectively, even under the most severe heat stress (44 °C). Pupae had relatively lower heat tolerance, with a survival rate of 72.51% at 44 °C. Although eggs exhibited a survival rate of 72.57% at 44 °C, the subsequent survival was significantly affected. The larval stage was the least heat-tolerant stage, with survival rates of 17.38% and 12.67% at 44 °C for the treated individuals and larval to adult period, respectively.

**Table 1:** Two-way ANOVA of the effects of temperature, developmental stage, and their interaction on the life parameters of *Ophraella communa*.

Parameter	Source^a^	df	Mean square	*F*	*P*
Survival rate	Temperature	3, 64	3058.69	362.39	<0.0001
	Stage	3, 64	5738.66	679.90	<0.0001
	Temperature × Stage	9, 64	453.35	53.71	<0.0001
Female proportion	Temperature	3, 48	179.48	29.60	<0.0001
	Stage	2, 48	1.41	0.23	0.7932
	Temperature × Stage	6, 48	12.71	2.10	0.0599
Fecundity	Temperature	3, 304	3689270.00	85.35	<0.0001
	Stage	3, 304	1256840.00	29.08	<0.0001
	Temperature × Stage	9, 304	448198.56	10.37	<0.0001
Female adult longevity	Temperature	3, 304	0.01	4.69	0.0033
	Stage	3, 304	0.19	121.69	<0.0001
	Temperature × Stage	9, 304	0.06	39.55	<0.0001
Male adult longevity	Temperature	3, 304	0.08	59.65	<0.0001
	Stage	3, 304	0.09	66.44	<0.0001
	Temperature × Stage	9, 304	0.10	72.10	<0.0001
F_1_ egg hatching rate	Temperature	3, 64	3490.22	194.58	<0.0001
	Stage	3, 64	42.05	2.34	0.0756
	Temperature × Stage	9, 64	26.16	1.46	0.1694

^a^Temperature levels are 28 (control), 40, 42, and 44 °C; the stages are egg, larva, pupa, and adult (for female proportion, only egg, larva, and pupa).

**Figure 2:**
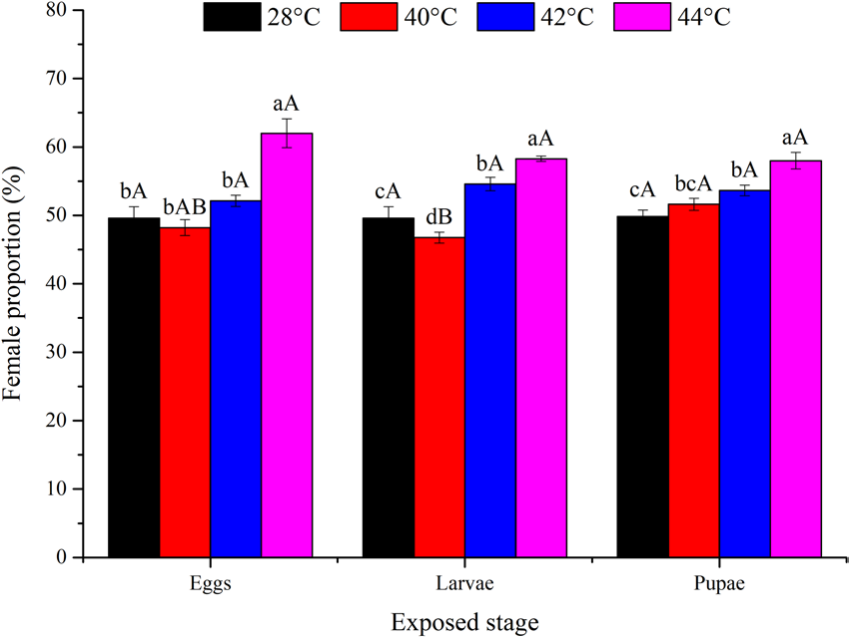
Mean ± SE female proportion [F/(F + M)] (%) of *Ophraella communa* when eggs, larvae, and pupae were exposed to high temperatures [28 (control), 40, 42 and 44 °C] for 3 h each day for 3, 5, and 5 days, respectively (by stage). The sample sizes for each treatment with five replicates were 2,000 (eggs), 1,800 (larvae) and 1,100 (pupae). Different lower-case and upper-case letters indicate significant differences among temperatures for the same life stage and among life stages at the same temperature level, respectively (Tukey’s HSD test, *P* < 0.05).

Overall, high temperatures ≥42 °C increased the female proportion. After exposure of the same developmental stage to 42 and 44 °C, the female proportions were significantly higher than those in the control treatment (except for eggs at 42 °C) (Fig. 2). Heat exposure during immature development resulted in a greater percentage of adult females regardless of the developmental stage exposed (*P* > 0.05; Table 1).

### Effects of high temperatures on fecundity, F_1_ egg hatching rate, and adult longevity

**Figure 3:**
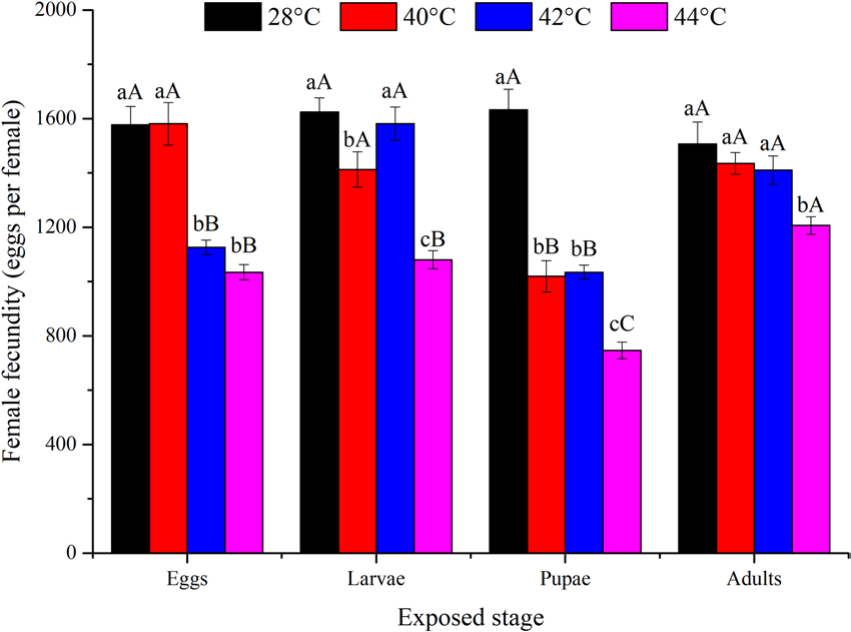
Mean ± SE female fecundity (number of eggs per female) of *Ophraella communa* when eggs, larvae, pupae, and adults were exposed to high temperatures [28 (control), 40, 42 and 44 °C] for 3 h each day for 3, 5, 5, and 5 days, respectively (by stage). The sample size for each treatment was 20 pairs, and each pair was treated as one replicate. Different lower-case and upper-case letters indicate significant differences among temperatures for the same life stage and among life stages at the same temperature, respectively (Tukey’s HSD test, *P* < 0.05).

**Figure 4:**
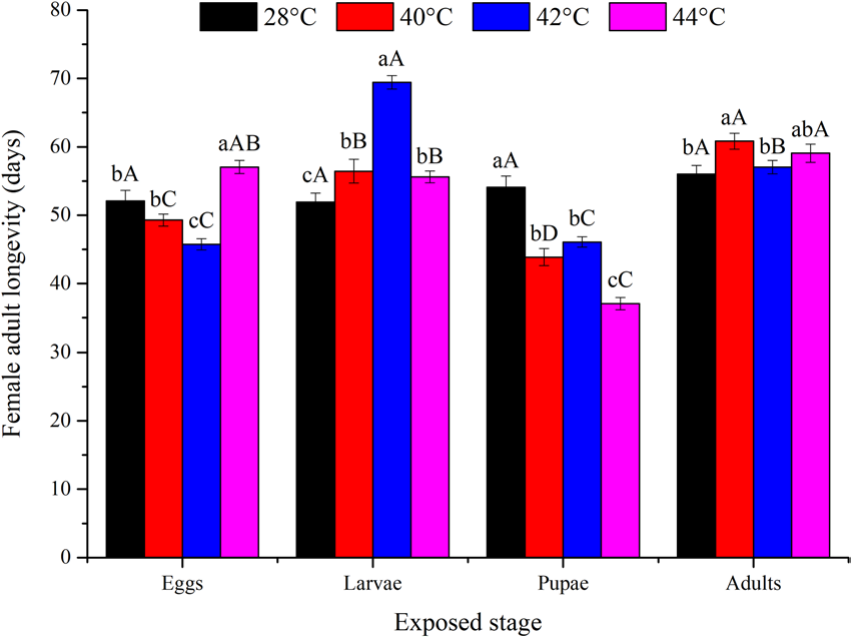
Mean ± SE female adult longevity (days) of *Ophraella communa* when eggs, larvae, pupae, and adults were exposed to high temperatures [28 (control), 40, 42 and 44 °C] for 3 h each day for 3, 5, 5, and 5 days, respectively (by stage). The sample size for each treatment was 20, and each individual was treated as one replicate. Different lower-case and upper-case letters indicate significant differences among temperatures for the same life stage and among life stages at the same temperature, respectively (Tukey’s HSD test, *P* < 0.05).

**Figure 5:**
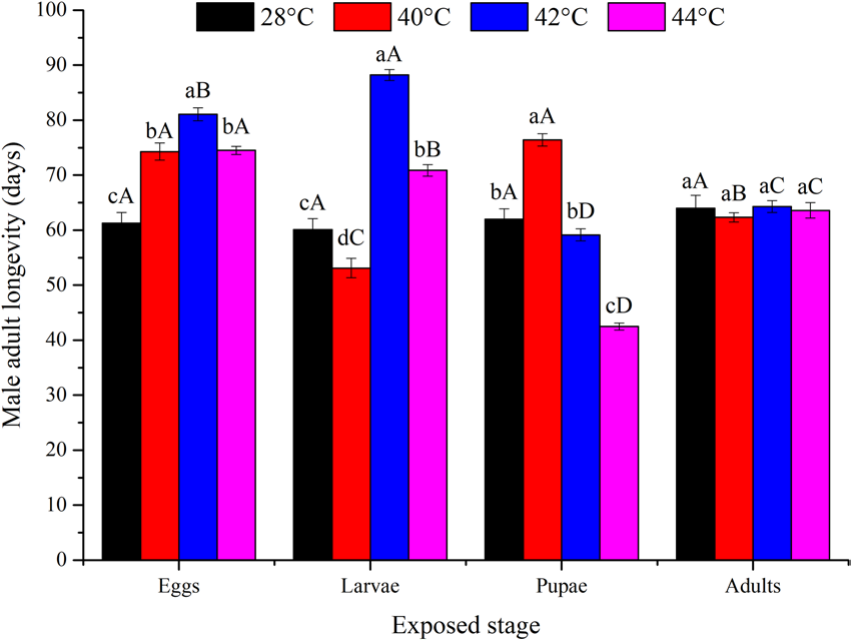
Mean ± SE male adult longevity (days) of *Ophraella communa* when eggs, larvae, pupae, and adults were exposed to high temperatures [28 (control), 40, 42 and 44 °C] for 3 h each day for 3, 5, 5, and 5 days, respectively (by stage). The sample size for each treatment was 20, and each individual was treated as one replicate. Different lower-case and upper-case letters indicate significant differences among temperatures for the same life stage and among life stages at the same temperature, respectively (Tukey’s HSD test, *P* < 0.05).

High temperature, the developmental stage exposed, and their interaction all had a significant influence on the fecundity of *O*. *communa* (*P* < 0.05; Table 1). Fecundity was significantly affected by the previous exposure of eggs (*F*_3,76_ = 38.07; *P* < 0.0001), larvae (*F*_3,76_ = 23.79; *P* < 0.0001), pupae (*F*_3,76_ = 67.60; *P* < 0.0001), and adults (*F*_3,76_ = 6.37; *P* = 0.0007) to high temperatures for short intervals. Fecundity was decreased at higher temperatures compared to the control (Fig. 3). Adults proved to be the most heat tolerant, with high egg production at any high temperature, especially at 42 and 44 °C. The pupal stage was the least heat tolerant, and female egg production was highly affected at any high temperature (Fig. 3). Adult longevity was significantly affected by temperature, the developmental stage exposed, and their interaction (*P* < 0.05; Table 1). Female adult longevity differed significantly among all temperatures when eggs (*F*_3,76_ = 27.94; *P* < 0.0001), larvae (*F*_3,76_ = 42.03; *P* < 0.0001), pupae (*F*_3,76_ = 43.80; *P* < 0.0001), or adults (*F*_3,76_ = 3.18; *P* = 0.0290) were exposed to high temperatures for 3 h daily intervals compared to the control (Fig. 4). Overall, the adult stage was the most heat-tolerant stage, with female adult longevity close to that in the control. Pupae were the least heat tolerant, with relatively shorter female adult longevity at any high temperature (Fig. 4). Male adult longevity also significantly differed among all temperatures when eggs (*F*_3,76_ = 37.73; *P* < 0.0001), larvae (*F*_3,76_ = 103.89; *P* < 0.0001), or pupae (*F*_3,76_ = 140.05; *P* < 0.0001) were exposed to high temperatures for 3 h daily intervals (Fig. 5). In total, male adult longevity was close to that in the control after the exposure of male adults to any high temperature, and there was no significant effect of the exposure of male adults to high temperatures (*F*_3,76_ = 0.37; *P* = 0.7732) (Fig. 5).

**Figure 6:**
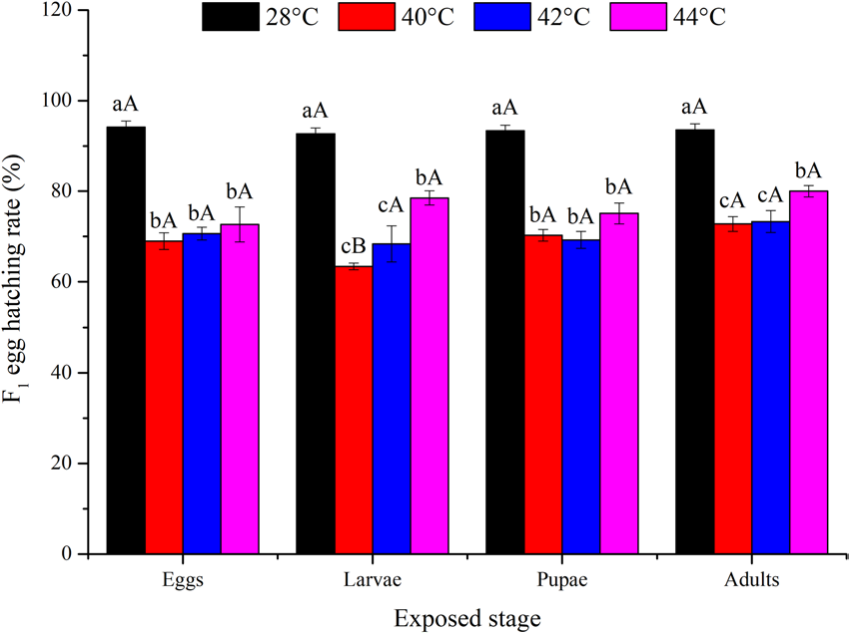
Mean ± SE percent of the F_1_ egg hatching rate of *Ophraella communa* when eggs, larvae, pupae, and adults were exposed to high temperatures [28 (control), 40, 42 and 44 °C] for 3 h each day for 3, 5, 5, and 5 days, respectively (by stage). The sample size for each treatment with five replicates was 1,600. Different lower-case and upper-case letters indicate significant differences among temperatures for the same life stage and among life stages at the same temperature, respectively (Tukey’s HSD test, *P* < 0.05).

The F_1_ egg hatching rate was significantly lower compared to the control (*P* < 0.05) when eggs (*F*_3,16_ = 46.25; *P* < 0.0001), larvae (*F*_3,16_ = 83.66; *P* < 0.0001), pupae (*F*_3,16_ = 53.34; *P* < 0.0001), or adults (*F*_3,16_ = 39.46; *P* < 0.0001) were exposed to high temperatures for short intervals (Fig. 6). However, the F_1_ egg hatching rate did not differ significantly among developmental stages at any temperature (*P* > 0.05; Table 1).

## Discussion

The negative impacts of extreme temperatures on insects depend on the intensity and duration of stress and the sex and stage of the insect^30^. In central and southern China, hot days are commonly observed under field conditions in summer, when the daily maximum air temperature can reach and sometimes exceeds 40 °C^19–21^. These hot conditions may be exacerbated by the effects of climate change^19–21^. As an overlapping-generation species with a relatively short generation period^31^, any developmental stage of *O*. *communa* might encounter a brief period of high-temperature stress; therefore, the developmental fitness of this beetle may be adversely affected when heat-sensitive life stages experience summer heat. Overall, our findings reflect that the intensity of the high temperature, the developmental stage, and their interaction all had significant influences on the life history parameters of *O*. *communa*.

### Survival under heat stress

In response to high temperatures, insects may die rapidly due to serious heat injury^27,33,34^. When the ambient temperature exceeds the upper temperature threshold, thermal stress sometimes leads to individual death or even local population extinction in insects^21,35,36^. In our experiments, the rapid response of eggs, larvae, pupae, and adults of *O*. *communa* to short intervals of high temperature exposure in the range of 42 to 44 °C was indicated by an immediate decline in survival. Rapid death under short-term heat exposure has also been reported in other beetles, including the Japanese beetle *Popillia japonica*^36^, the tamarisk leaf beetle *Diorhabda carinulata*^37^, and the ladybird beetle *C*. *montrouzieri*^25^.

Subsequent survival was significantly affected when eggs were exposed to ≥40 °C or larvae were exposed to ≥42 °C, suggesting that heat stress could affect survival as a result of thermal injury. Carry-over effects have been reported to occur during later developmental stages and result in reduced subsequent survival duration^14^.

In our study, daily exposure to 40 °C for 3 h did not significantly reduce egg or pupal survival. Even at the highest temperature (44 °C), the survival rates of eggs, pupae and adults of *O*. *communa* were over 50%, and the survival rate of *O*. *communa* larvae was close to 20%, these results indicated that *O*. *communa* has a large thermal tolerance plasticity. These high survival rates may relate to recovery from heat injury during the cooler period between high temperatures or insufficient damage to affect survival^38^, which is common in insects^38,39^. It is believed that recovery times between high temperatures can allow injuries to be repaired, as aphids were shown to exhibit higher protein and osmolyte levels in a repeated exposure treatment compared to a prolonged exposure treatment group^39^. One possible explanation is that the proteins and osmolytes were upregulated during the recovery period^39^.

The survival rates of adult females were significantly higher compared to those of males in our study. These results agree with the conclusions of previous studies that showed that adult females of the whitefly *T*. *vaporariorum* and the aphid parasitoid *Aphidius avenae* were more resistant to heat injury than males^23,35^. Esperk *et al*. found that the heat tolerance of *Sepsis punctum* was positively affected by body size^40^. A large body size is thought to help prevent dehydration, which might partly explain the greater tolerance of females to heat stress^20,21^. The larger female adult body size compared to that of adult males under heat stress^20^ may partly explain the higher thermotolerance of female adult *O*. *communa*.

### Female proportion under heat stress

The present study showed that the female proportion of *O*. *communa* at ≥42 °C was higher than that at room temperature (28 °C). This contrasts with the results for several other insect species^23,41–43^. However, in a few studies, the female proportion increased with temperature, as was the case for the parasitoid *Campoletis chlorideae*^44^ under controlled temperatures. Differential mortality between males and females in *Trichogramma euproctidis*^45^ and *Aphidius gifuensis*^46^ may be caused by developmental temperatures that result in a biased sex ratio, which might explain this phenomenon. The influence of extreme variations in temperature on sex ratio was reported by Werren and Charnov^47^, who predicted that there may be sex-biased mortality during a cold or heat wave. The high female proportion in *O*. *communa* could be due to the male-biased immature mortality under heat stress. Temperature not only affects the sex ratio of the current generation but also the sex ratio of the offspring^27,45^. The female-biased sex ratio in *O*. *communa* may increase its population thermal tolerance and will help this beetle rapidly re-establish its populations.

### Effects of heat stress on reproduction

An improvement in thermotolerance usually occurs at the cost of negative physiological consequences^48^. Therefore, a trade-off between survival and reproductive output is widely present in insects under stressful conditions^48,49^. On the other hand, in response to high temperatures, insects may die slowly because injurious effects accumulate slowly and are displayed at later stages of development^27,33,34^. Even if some individuals are able to survive after brief heat stress, their fitness is often reduced^21^. It has been reported that reproductive traits are more sensitive to thermal stress than other physiological traits^50^; thus, high temperatures experienced during early life stages are carried over to affect adult reproduction^32^. The present results showed that the fecundity of *O*. *communa* females was significantly affected by the 3 h of exposure of eggs and adults to 42 and 44 °C, larvae to 40 and 44 °C, and pupae to 40, 42 and 44 °C. Our previous study indicated that the fecundity of *O*. *communa* adults significantly decreased with temperature when the temperature exceeded the optimal range during 2 h of controlled heat shock^3^, which was consistent with the present findings. The adverse effect of high temperatures on this beetle’s fecundity may affect its field population and thus its ability to control common ragweed. These results were similar to those of other studies on the wasp *A*. *avenae*^35^, *D*. *carinulata*^37^, the bollworm *Helicoverpa armigera*^33^, the leafminer *Liriomyza huidobrensis*^51^, the flesh fly *Sarcophaga crassipalpis*^52^, the fruit fly *Drosophila buzzatii*^50^ and the whitefly *T*. *vaporariorum*^23^. In some insect species, high temperatures disrupt the functioning of the reproductive system in both sexes^27^. A major effect of heat shock on male fecundity is direct injury to the sperm. For example, no sperm was present in the spermatheca of *S*. *crassipalpis* females mated with heat-shocked males^52^. The reduction in male fertility caused by heat shock was consistent with findings for the parasitic wasp *T*. *euproctidis*^45^ and the butterfly *Bicyclus anynana*^53^. Generally, male ejaculate contains both sperm and male-derivate substances, and the substances can modify the female’s behavior and physiology and may be used for somatic maintenance or fecundity enhancement^54^. Other studies revealed that the effect of heat shock on female fecundity is likely due to direct injury to developing oocytes^52^, and a decrease in female fertility caused by heat shock is likewise described in *T*. *euproctidis*^45^. On the other hand, heat stress greatly decreased the frequency of courtship and mating by reducing the attractiveness of males to females in three *Drosophila* species (*D*. *melanogaster*, *D*. *simulans* and *D*. *mojavensis*)^55^ and the diamondback moth, *P*. *xylostella*^49^. It is reported that *O*. *communa* females laid fewer eggs with short mating time, and the copulation time was decreased with increasing temperature^56^. Multiple mating (female acceptance of copulations with different males (polyandry) or repeated copulations with the same male (monogamy)) has a positive effect on egg production has been reported in many insects^57–59^. *Ophraella communa* adults mate many times throughout their lifespan and even mate several times in one day, its fitness parameters are positively associated with the number of copulation events, and multiple-mating behaviour increase the fitness benefits^60^. Adult females of *O*. *communa* may mate with multiple males in a lifetime in the field due to their strong activity, our methods (repeated copulations with the same male) may underestimate the real reproduction of *O*. *communa*. Meanwhile, the replacement of earlier died males with other males of the similar age and treatment will change the numbers of mated males, which will influence the results. We also suggests that the effect of mating patterns under heat stress to address in future. The mechanisms of the decrease in reproductive output after temperature stress may be due to impaired oocyte development, decreased mating success, sperm production, sperm viability^61^, and changed mating patterns^58^.

### Heat stress effects on lifespan

In general, a straightforward trade-off between damage repair and somatic maintenance could reduce longevity^61^. Our previous study showed that the longevity of *O*. *communa* adults significantly decreased after the exposure of adults to high temperatures ≥35 °C for 2 h^3^, which was consistent with this general principle. However, overall, heat stress had no significant effect on the lifespan of adult *O*. *communa* after the exposure of adults to high temperatures ≥40 °C for 3 h in the present study, which differed from the previous results. Mild temperature hardening in nature can increase insects’ thermotolerance^51^; thus, the time at which insect samples are collected from the field also influences adult longevity under heat stress. Some previous studies also reported that heat stress increased the longevity of some *Drosophila* species^62^, such as *D*. *melanogaster* males (exposure of adults to 34 °C for 3 h)^63^, parasitoids, such as females of the wasp *A*. *avenae* (exposure of adults to 36 °C for 1 h)^35^, and the oriental fruit moth, *Grapholita molesta* (exposure of adults to 38 °C for 4 h)^64^. In our study, there was no obvious change trend in adult *O*. *communa* longevity after exposure of the preadult stages to high temperatures ≥40 °C for 3 h, and an increase or decrease may be caused by chance. Different methods of heat exposure may have different levels of among-stages and species-related variance. Fluctuating high temperatures provided the chance for surviving insects to improve their heat tolerance and fitness, including longevity^65,66^. Thus, the lifespan of *O*. *communa* may be longer in the field in summer heat than that under constant high-temperature conditions in the lab.

### Heat stress effects on progeny

The fitness of offspring might also be affected by high maternal temperatures^49^. The F_1_ egg hatching of *O*. *communa* was likewise directly proportional to the high temperatures in our study, which indicated that the effects of heat shock could be transferred to the next generation^27^. Previous studies found that F_1_ egg-hatching rates were affected by high temperature in the whitefly *T*. *vaporariorum*^23^ and the fruit flies *D*. *melanogaster*^67^ and *S*. *crassipalpis*^52^. Extreme examples showed that heat stress affected *T*. *euproctidis* and *S*. *crassipalpis* males, and this resulted in no eggs being fertilised^45,52^. Male sterility or reduced fertility caused by heat stress was likewise found in *D*. *buzzatii*, and this also affected progeny fitness^50,68^. The decrease in the egg hatching rates in the next generation of *O*. *communa* after exposure of different life stages to high temperatures in this study might be due to male infertility.

### Stage-specific heat effects

Stage-specific heat tolerance has been observed in many insects^14,19,32,52,65^. The life history traits of *O*. *communa* were also affected by its different developmental stages being exposed to heat stress in our study. Normally, less mobile stages (like eggs and pupae) are more resistant to heat than mobile stages (like larvae and adults)^30^. By contrast, from the standpoint of survival, in *O*. *communa*, the larval stage was the most heat-susceptible life stage, and the adult stage was the least heat-susceptible life stage in our study. The relative sensitivity of less mobile stages was observed in *P*. *xylostella* (eggs and pupae)^19^ and *Wyeomyia smithii* (pupae)^69^, and the relative insensitivity of mobile stages was also observed in *Tenebrio molitor* (adults)^70^. Stress resistance may be affected by past selection pressures depending on the environments in which the different developmental stages are found^19^. *Plutella xylostella* eggs and pupae usually occur on the underside of leaves, where temperatures are cooler on hot days, and this may help explain the relative sensitivity of these stages compared to the pattern in other insects^19^. *Ophraella communa* prefers to lay eggs on the back of the mid and basal leaves, and first instar larvae stay for several hours near the egg shell (personal observation), which may contribute to our understanding of the high immediate and subsequent death of eggs and first instar larvae. However, overall, the female proportion and F_1_ egg hatching rate of *O*. *communa* were not significantly affected by the developmental stage exposed. The fecundities and adult longevities of *O*. *communa* appeared to be more depressed by heat stress during the pupal stage than during other stages. We assume that adults are more heat tolerant than other stages based on their high survival rate, high female fecundity, longevity that is similar to control adult longevity and relatively high F_1_ egg hatching rate. *Ophraella communa* overwinters and expands its distribution mainly in the adult stage^9^, suggesting that adults are better able to tolerate environmental stress than other stages. Mobile adults likely experience a greater range of thermal microclimates, and greater variability in tolerance or greater basal (innate) tolerance might be expected^28^. The greater mobility of larvae and adults compared to other stages allows them to search for low-temperature microclimates to reduce thermal injury through behavioural thermoregulation^71^, which increases the thermal tolerance of larvae and adults in the field. The stage-specific heat tolerance of *O*. *communa* is beneficial for the establishment and expansion of this natural enemy in the field.

### Potential application in biological control

Temperature is not constant in the field, which varies over time. The prior experience of natural conditions in the field could improve the heat tolerance of some insects^16^. Ectotherms exposed to daily thermal fluctuations usually showed higher upper thermal limits than those exposed to constant temperature conditions^72^, which implies that they may be able to survive in the field at higher temperatures than predicted from laboratory experiments conducted under constant temperatures^61^. The field microclimates experienced by each life stage can be used to inform the avoidance of extreme temperatures^70^, which will increase insects’ thermal tolerance in the field. Likewise, the results from our constant temperature model might underestimate the thermal tolerance of *O*. *communa* in the field. Meanwhile, humidity is also an important abiotic factor influencing the biology of insects. The effects of temperature depend on the relative humidity (RH) level^73^, and RH also changes with time. Therefore, the life history parameters of *O*. *communa* in environments in which thermal and RH environments fluctuate need to be investigated in future studies. Many of the changes in insect development and reproduction may result from changes in the endocrine system^22^, and insects must constantly adjust their physiologies to changing thermal conditions^61^. The physiological mechanisms and the secondary sex ratio of *O*. *communa* in response to heat stress should be further studied to improve the use of this biological control agent. The results indicate that *O*. *communa* can tolerate 44 °C heat for up to 3 hours, which may contribute to its expansion into the lower latitudes in China, where its host (common ragweed) is widely distributed. We conclude that *O*. *communa* possesses a degree of heat tolerance that allows it to survive on hot days in summer.

## Materials and Methods

### Host plants

*Ambrosia artemisiifolia* seeds were collected from more than ten thousand plants in the town of Dajing (28°56′26″N, 113°14′38″E) in Miluo County, Yueyang City, Hunan Province, China, in late October 2010^74^. The seeds were then stored at 4 °C. Adequately stored seeds were germinated in a greenhouse in late March 2011, and when the seedlings reached a height of approximately 15 cm^74^, some of them were used in adult heat treatments and tests of longevity, fecundity, and F_1_ egg hatching in *O*. *communa*. The apical buds of the remaining seedlings were removed to prevent apical dominance, and the seedlings were transplanted into pots (21 × 17 cm) containing soil at one seedling per pot. One thousand pots containing treated common ragweed seedlings were prepared and placed in a greenhouse. All the plants were watered in a timely manner and fertilised (N:P:K = 13:7:15) twice per month to maintain normal growth^31^. The potted plants were used in the heat treatments of eggs, larvae and pupae when the plants were approximately 40 cm high.

### Insects

More than 1,000 *O*. *communa* adults were collected from the town of Dajing (28°56′26″N, 113°14′38″E) in Miluo County, Yueyang City, Hunan Province, China, on June 24, 2011. Colonies of the beetle were maintained on *A*. *artemisiifolia* plants under natural light in a greenhouse at 28 ± 2 °C at the Institute of Plant Protection, Hunan Academy of Agricultural Sciences (25°21′18″N, 114°33′40″E), Changsha, Hunan Province, China^74^.

Six pairs of *O*. *communa* adults were randomly collected from the rearing colony and placed with the aid of a fine brush (size 0) onto a pot containing a fresh common ragweed plant, which was then covered with nylon gauze (40 mesh size). After a 2-d oviposition period, the beetles were removed to synchronise the development of stages for exposure to the thermal treatments. Approximately 400 plants were prepared for the following high-temperature stress treatments.

### Thermal treatments

The duration and intensity of heat stress were based on the duration and intensity of high temperatures in summer, which are usually a few hours of particularly high temperatures in central China (max temperature 44 °C for approximately 3 h per day for 3–5 consecutive days)^20^. The treatments examined the effects of high temperature (40, 42, and 44 °C) on beetle life history parameters using periods of exposure of 3 h per day for 3, 5, 5, and 5 days for eggs, larvae, pupae, and adults, respectively. Control insects were kept at 28 °C to allow normal *O*. *communa* development^31^. The exposure periods were determined based on the developmental periods of the different developmental stages of *O*. *communa* (4.0 days for eggs, 7.6 days for larvae, and 6.0 days for pupae) obtained at a constant high temperature (32 °C) in an earlier laboratory bioassay^31^ and the hottest days (up to 44 °C) that occur for a duration of 3–5 days in Changsha, Hunan Province, China^20^. The experiments were conducted in early to mid-July 2011 (the field temperature during July in Changsha was 23–40 °C, with an average of 31.5 °C). The high-temperature exposure treatments for each treatment were performed separately in environmental chambers (PRX-450D, Ningbo Haishu Safe Experimental Equipment Co. Ltd., Zhejiang, China) at 28 (untreated control), 40, 42, or 44 ± 1 °C, with a RH of 70 ± 5%. The optimal RH at 25 °C for the development of *O*. *communa* in the laboratory ranges from 75% to 90%^31^. In recent years, the RH in Changsha fluctuated around 70% in summer (personal observation). Therefore, we selected 70% RH as the experimental condition. The exposure treatments were also conducted under a photoperiod of 14:10 (L:D) h^31^ and a light intensity of 12,000 LX for 3 h daily for 3 or 5 consecutive days.

### Effects of high temperatures on survival and female proportion

One hundred eggs ≤12 h old, 90 first instar larvae ≤24 h old, and 55 pupae ≤24 h old were separately retained on three potted plants. Twenty ragweed plants were used for each developmental stage, and they were then exposed to high temperatures in environmental chambers, after which the infested potted plants were kept in a greenhouse. A total of 240 ragweed plants were used. Following the high-temperature stress treatments, the treated pupae were collected by detaching the leaves on which they occurred and placing the individual leaves into open transparent plastic boxes (19 × 12 × 6 cm) in an unsealed plastic cuvette tube covered with nylon gauze (60 mesh size) in the laboratory at 28 ± 2 °C and 70 ± 5% RH, where the pupae were checked daily for adult emergence. The treated eggs and larvae were kept in a greenhouse until they reached the pupal stage. The process for these pupae was the same as that for the treated pupae following the high-temperature stress treatments. The sex of each newly emerged adult was determined using a stereomicroscope, and the female proportion was calculated. The survival (in days) of male and female adults was recorded for each temperature treatment.

Newly emerged adults ≤24 h old (125 pairs) were randomly selected from the rearing colony for exposure to high temperatures. Each adult pair was released onto a fresh ragweed seedling (15 cm height) in a plastic box (19 × 12 × 6 cm) with a hole (15 × 4 cm) covered with nylon gauze (60 mesh size). The survival of male and female adults was checked daily.

The survival rates for eggs, larvae, and pupae were determined using the following equation: (number of emerged individuals of the next stage)/(number individuals in the treated stage) × 100%. The survival rate of adults was determined using the following equation: (number of survived adults)/(number of treated adults) × 100%. The subsequent survival rates of treated eggs and larvae were determined using the following equation: (number of emerged adults)/(number of individuals in the treated stage) × 100%. The female proportion was determined using the following equation: (number of females)/(total number of females and males) × 100%.

### Effects of high temperatures on adult longevity, fecundity, and F_1_ egg hatching

Once the test insects reached the adult stage, insects for which different life stages had been exposed to a range of high temperatures as described above were evaluated for fecundity and adult longevity in the greenhouse. To measure fecundity and longevity, each pair of adults was placed on a fresh potted ragweed seedling in a plastic box (19 × 12 × 6 cm) with a hole (15 × 4 cm) covered with nylon gauze (60 mesh size), with twenty boxes treated as experimental replicates^31^. The number of eggs laid by the females and the duration of adult survival were recorded daily until all adults died. For each treatment, 1,600 eggs were retained on 20 seedlings to evaluate the egg hatching rate in the greenhouse. Other eggs were removed after counting, and the seedlings were changed when necessary. Egg viability was estimated based on the number of emerged larvae. If a male died, then another treated male of approximately the same age was added (the longevity of these males was not recorded)^31^.

### Statistical analyses

Data were checked for normality and homoscedasticity and, if needed, were arcsine square root or log transformed. All data were analysed using SPSS 21.0 (SPSS Inc., Chicago, Illinois, USA). The survival rate, female proportion, and F_1_ egg hatching rate (%) were arcsine square root transformed, and adult longevity was transformed using log_10_ (x + 1) before analysis^31^. The data were subjected to two-way analysis of variance (ANOVA) to test the effects of temperature, the stage exposed to the heat treatment, and their interaction on the life history parameters of *O*. *communa*. Means were separated using Tukey’s HSD (honestly significant difference) test (one-way ANOVA) when significant differences were found at *P* < 0.05 and were denoted as the means ± SE (standard error of the mean).
